# Tension Force-Induced ATP Promotes Osteogenesis Through P2X7 Receptor in Osteoblasts

**DOI:** 10.1002/jcb.24863

**Published:** 2014-11-11

**Authors:** Taro Kariya, Natsuko Tanabe, Chieko Shionome, Soichiro Manaka, Takayuki Kawato, Ning Zhao, Masao Maeno, Naoto Suzuki, Noriyoshi Shimizu

**Affiliations:** 1 Nihon University Graduate School of Dentistry, TokyoJapan; 2 Department of Biochemistry, Nihon University School of DentistryTokyo, Japan; 3 Division of Functional Morphology Dental Research Center, Nihon University School of DentistryTokyo, Japan; 4 Department of Orthodontics, Nihon University School of DentistryTokyo, Japan; 5 Department of Oral Health Sciences, Nihon University School of DentistryTokyo, Japan; 6 Department of Endodontics School of Dentistry, Shandong UniversityJinan, Shandong Province, China

**Keywords:** TENSION FORCE, P2X7, ATP, OSTEOGENESIS

## Abstract

Orthodontic tooth movement induces alveolar bone resorption and formation by mechanical stimuli. Force exerted on the traction side promotes bone formation. Adenosine triphosphate (ATP) is one of the key mediators that respond to bone cells by mechanical stimuli. However, the effect of tension force (TF)-induced ATP on osteogenesis is inadequately understood. Accordingly, we investigated the effect of TF on ATP production and osteogenesis in MC3T3-E1 cells. Cells were incubated in the presence or absence of P2X7 receptor antagonist A438079, and then stimulated with or without cyclic TF (6% or 18%) for a maximum of 24 h using Flexercell Strain Unit 3000. TF significantly increased extracellular ATP release compared to control. Six percent TF had maximum effect on ATP release compared to 18% TF and control. Six percent TF induced the expression of Runx2 and Osterix. Six percent TF also increased the expression of extracellular matrix proteins (ECMPs), ALP activity, and the calcium content in ECM. A438079 blocked the stimulatory effect of 6% TF on the expression of Runx2, Osterix and ECMPs, ALP activity, and calcium content in ECM. This study indicated that TF-induced extracellular ATP is released in osteoblasts, suggesting that TF-induced ATP promotes osteogenesis by autocrine action through P2X7 receptor in osteoblasts. J. Cell. Biochem. 116: 12–21, 2015. © 2014 The Authors. Journal of Cellular Biochemistry published by Wiley Periodicals, Inc.

The skeletal system is developed and adapted by hormones, cytokines, and external factors such as mechanical stimuli. The bone mass of adults is maintained by a dynamic process involving a balance of bone remodeling. Mechanotransduction is a major factor that regulates skeletal mass [Robling et al., 2006b; Maeno et al., 2013].

Nucleotides, such as ATP, are key mediators that respond to mechanical stimuli in bone cells [Dixon and Sims, 2000; Robling et al., 2006a]. Extracellular ATP is also stress-responsive. ATP production induced by mechanical stimulation is a cell-regulated process that occurs upon the cell lysis [Romanello et al., 2001; Genetos et al., 2005; Riddle et al., 2007; Praetorius and Leipziger, 2009]. ATP release is induced by mechanical stresses such as stretching, shearing, media changes, and osmotic stress in various cell types [Praetorius and Spring, 2001]. The release of ATP induced by mechanical stimulation-induced ATP release is correlated closely with the elevation in intracellular Ca^2+^ concentrations ([Ca^2+^]_i_). It also suggests that Ca^2+^-dependent exocytosis may play a role in ATP production in some cell types [Boudreault and Grygorczyk, 2004]. P2 receptors are expressed in bone cells including osteoblasts and osteoclasts. P2 receptors can be divided into the P2Y family of G protein-coupled receptors and the P2X family of ligand-gated cation channels [Burnstock, 2007]. Nucleotides are released by mechanical stimulation or inflammation and may serve as autocrine/paracrine mediators of both osteoblast and osteoclast function [Jorgensen et al., 2002; Hoebertz et al., 2003; Genetos et al., 2005]. Osteoblasts express both P2X and P2Y receptors [Dixon and Sims, 2000; Hoebertz et al., 2000]. P2X7 receptor promotes dye uptake in response to fluid shearing stress in mouse calvarial cells [Li et al., 2005]. Thus, these reports suggest that pore formation may play a role in mechanotransduction [Grol et al., 2009]. P2rx7^−/−^ mice were found to exhibit decreased periosteal bone formation in long bones but length was not affected [Ke et al., 2003]. Moreover, P2rx7^−/−^ mice were showed reduced osteogenesis in response to mechanical loading [Li et al., 2005]. These reports suggest that the P2X7 receptor may play a role in skeletal remodeling and mechanotransduction.

During orthodontic tooth movement, the mechanical activities of the extracellular matrix of periodontal tissues determine the cellular processes that regulate the remodeling of the periodontal ligament and alveolar bone [Krishnan and Davidovitch, 2006]. For example, the traction side induces bone formation in orthodontic tooth movement. However, the effects of tension force (TF)-induced ATP on osteogenesis by osteoblasts remains elusive.

In this study, we found that TF induced the release of ATP, which mediated the activation of P2X7 receptor, and increased osteogenesis in osteoblasts. This novel mechanism explains, in part, the stimulatory effects of TF in promoting bone formation in vivo.

## MATERIALS AND METHODS

### CELL CULTURE

MC3T3-E1 cells from a mouse calvarial cell line were obtained from Riken Bio Resource Center (Tsukuba, Japan) and used as osteoblasts. Cells were maintained in α-minimal essential medium (α-MEM; Gibco BRL, Rockville, MD) containing 10% (v/v) heat-inactivated fetal bovine serum (HyClone Laboratories, Logan, UT) and 1% (v/v) penicillin–streptomycin solution (Sigma–Aldrich, St. Louis, MO) at 37°C in a humidified atmosphere of 95% air and 5% CO_2_. The medium was changed every 3 days. Cells were plated on flexible-bottomed culture plates (Flexcell Corp., Hillsborough, NC) at a density of 2 × 10^4^ cells/cm^2^ onto six-well plates.

### APPLICATION OF TENSION FORCE

Briefly, cyclic TF was applied on MC3T3-E1 cells with the Flexercell Strain Unit (FX-3000, Flexcell Corp.) [Shimizu et al., 1995]. The Flexercell Strain Unit was used in order to mechanically strain cells. MC3T3-E1 cells were seeded on flexible-bottomed six-well plates that have a hydrophilic surface at a density of 2 × 10^4^ cells/cm^2^, then cells were placed onto a vacuum manifold controlled by computer software and a solenoid valve. The system uses a vacuum source to apply a negative pressure causing a downward deformation of the membrane to which the cells are attached. The strain applied over the loading-post regions is approximately equal in the radial and circumferential directions [Vande Geest et al., 2004]. Cells were flexed at 6 cycles/min (5 s strain, 5 s relaxation) with 6% or 18% TF for maximum 24 h. We selected the same TF strength as referenced elsewhere [Shimizu et al., 1995]. Controls were prepared in an identical manner and cultured on unstrained flexible-bottomed plates.

### LUCIFERIN–LUCIFERASE BIOLUMINESCENCE ASSAY

Cells were plated at a density of 2 × 10^4^ cells/cm^2^ onto six-well plates. The medium was replaced with 3 ml of fresh culture medium containing ARL67156 − ATPase inhibitor (Sigma–Aldrich) after plating for 24 h. Cells were then incubated for 30 min. TF stimulation was obtained by 6% or 18% TF for a maximum of 15 min. All incubations were performed at 37 °C, 5% CO_2_. Particular care was taken to minimize mechanical perturbation of cells during these procedures. The extracellular ATP concentration was determined using Kinsiro ATP Luminescence kit (Toyo Ink SC Holdings Co., Tokyo, Japan). Fifty microliters of the luciferin-luciferase assay medium, dissolved in culture medium, was added to a 50 μl sample. The resulting light signal was immediately measured by a Luminescencer Octa from ATTO (ATTO Co., Ltd., Tokyo, Japan). A calibration curve was generated for each luciferase assay using serial dilution of an ATP standard. All reagents used to stimulate cells were tested in control experiments. Statistical differences among groups were evaluated by unpaired *t*-test and n showed the number of independent preparations done in triplicate.

### REAL-TIME POLYMERASE CHAIN REACTION (REAL-TIME PCR)

MC3T3-E1 cells were plated into six-well microplates and cultured with medium for up to 14 days. Total RNA was isolated from the cultured cells on days 3, 7, and 14 of culture using an RNeasy Mini Kit (Qiagen, Hilden, Germany). The amount of RNA was measured using NanoDrop 1000 (ND-1000; Thermo Fisher Scientific, Wilmington, DE). Complementary DNA (cDNA) was synthesized from 0.5 μg of DNase-treated total RNA using the PrimeScript RT reagent kit, and the resultant cDNA was subjected to real-time PCR using the SYBR Green I dye. The reactions were performed in a 25-μl total volume containing 12.5 µl of a SYBR premixed Ex *Taq* (Takara Bio, Shiga, Japan), 0.5 μl (20 mM) of each sense and antisense primers (Table1), 9.5 μl of dH_2_O, and 0.5 μg/2 μl of cDNA. The PCR assays were performed on a Smart Cycler (Cepheid, Sunnyvale, CA) and analyzed using Smart Cycler software. The PCR protocol consisted of 35 cycles at 95°C for 5 s and 60°C for 20 s. All real-time PCR experiments were performed in triplicate, and the specificity of the PCR products was verified by a melting curve analysis. Calculated values for gene expression levels were normalized to the levels of glyceraldehyde 3-phosphate dehydrogenase (GAPDH) mRNA [Kuwabara et al., 2011].

**I:** PCR Primers Used in the Experiments

Target	Primers	GenBank Acc.
Runx2	5′-CACTCTGGCTTTGGGAAGAG-3′	NM_001145920.1
	5′-GCAGTTCCCAAGCATTTCAT-3′	
Osterix	5′-GGTAGGCGTCCCCCATGGTTT-3′	NM_130458.3
	5′-AGACGGGACAGCCAACCCTAG-3′	
P2X7	5′-TGCAGCTGGAACGATGTCTTG-3′	NM_011027.2
	5′-CGCTGGTACAGCTTATCGCTCA-3′	
Type I collagen	5′-TGGGCGCGGCTGGTATGAGTTC-3′	NM_007743.2
	5′-ACCCTGCTACGACAACGTGCC-3′	
BSP	5′-AATTCTGACCCTCGTAGCCTTCATA-3′	NM_008318.3
	5′-GAGCCTCGTGGCGACACTTA-3′	
OPN	5′-TACGACCATGAGATTGGCAGTGA-3′	NM_009263.3
	5′-TATAGGATCTGGGTGCAGGCTGTAA-3′	
OCN	5′-AAGCAGGAGGGCAATAAGGT-3′	NM_007541.2
	5′-ACCCTGCTACGACAACGTGCC-3′	
GAPDH	5′-AAATGGTGAAGGTCGGTGTG-3′	NM_008084.2
	5′-TGAAGGGGTCGTTGATGG-3′	

### SDS–PAGE AND WESTERN BLOTTING

After TF stimulation, cells were cultured in differentiation medium without FBS for an additional 24 h. The cells were collected and subjected to sodium dodecyl sulfate–polyacrylamide gel electrophoresis (SDS–PAGE) on 10% polyacrylamide gels (8.3 cm × 6.5 cm × 0.75 mm) using a discontinuous Tris–glycine buffer system. Media samples containing 10 μg of extracellular protein were dissolved in 10 μl of sample buffer containing 1% SDS, 2 M urea, 15 mg/ml dithiothreitol, and bromophenol blue, and then heated at 95°C for 5 min before loading onto the gel. Gels were run at 150 V for 60 min. Gel-separated proteins were transferred to a membrane using a semidry electrotransfer unit with a continuous buffer system consisting of 39 mM glycine, 48 mM Tris, 0.0375% SDS, and 20% (v/v) methanol at a constant amperage of 0.8 mA/cm^2^ for 60–90 min. On completion of the transfer, the transfer membrane was treated with 25% (v/v) blocking reagent in Tris-buffered saline (TBS) (10 mM Tris, 145 mM NaCl, pH 7.4) at 4°C for 18 h. The sheet was washed in TBS containing Tween-20 (TBS–Tween) and then incubated at room temperature for 90 min with rabbit biotin-labeled polyclonal IgG antibodies against Runx2, Osterix, and β-tubulin (all from Santa Cruz Biotechnology, Santa Cruz, CA). Primary antibodies were diluted 1:200 in distilled water containing 10% (v/v) blocking reagent. β-Tubulin was used as an internal standard. The membranes were washed in TBS–Tween and incubated at room temperature for 60 min with the appropriate biotin-conjugated secondary antibodies that were diluted 1:10,000 in distilled water containing 10% blocking agent. The membranes were washed in TBS–Tween and phosphate-buffered saline (PBS; Nissui Pharmaceutical Co. Ltd., Tokyo, Japan) and then incubated for 30 min at room temperature with horseradish peroxidase-conjugated streptavidin diluted with PBS. Immunoreactive proteins were visualized using a commercial chemiluminescence kit (Amersham Life Sciences, Buckinghamshire, UK) and autoradiography with X-ray film (Eastman Kodak, New Haven, CT). As a control, membranes were exposed to diluted normal rabbit serum; the dilution factor was the same as that used for the primary antibodies. Pre-stained molecular weight standards were run on the same gel. The blot intensity was quantified by digital image analysis software (GS-800 Calibrated Densitometer and Quantity One Version 4.2.1, Bio-Rad, Hercules, CA). n showed the number of separate experiments done in triplicate.

### ENZYME-LINKED IMMUNOSORBENT ASSAY (ELISA)

After TF stimulation, cells were cultured in differentiation medium without FBS for an additional 24 h. The amount of type I collagen, BSP, OPN, and OCN in the culture medium at 3, 7, and 14 days was determined using a commercially available ELISA kit (R&D Systems, Cusabio Biotech Co, Uscn Life Science, Inc. and Biomedical Technologies, Inc.) according to the manufacturer's instructions. Assays were performed in triplicate, in three independent preparations.

### ALP ACTIVITY AND MINERAL CONTENT IN MATRIX

Cells were fixed with 4% neutral buffered formalin overnight at 4°C. ALP activity was revealed using a mixture of 0.25 mM naphthol AS-MX phosphate (Sigma–Aldrich) and 1.25 mM Fast Red B salt (Sigma–Aldrich) in 0.1 M Tris–HCl, pH 8, for 1 h in the dark. Cells were observed using a microscope with a Plan ×10 DL objective (Nikon). To quantify mineral content, cells were cultured with culture medium containing 50 μg/ml ascorbic acid and 10 mM β-glycerophosphate [Roth et al., 1999]. The culture medium was changed every 2–3 days. After 14 days, the medium was discarded; 300 ml of 0.5 M HCl was added to each well, and the cells were left overnight to decalcify the mineralized nodules. The calcium content was determined quantitatively using a Calcium E-Test Wako (Wako Fine Chemicals, Osaka, Japan) following the manufacturer's instructions. The protein content was determined quantitatively using the Bio-Rad protein assay solution (Bio-Rad Laboratories, Hercules, CA) after the evaporation of HCl from samples. Assays were performed in triplicate, in three independent preparations.

### STATISTICAL ANALYSIS

All experiments were performed in triplicate or quintuplicate, n = 3 independent preparations. Each value represents the mean ± standard error (SEM). The significance of differences was determined using one-way analysis of variance followed by Tukey's multiple comparisons test or two-way analysis of variance by Bonferroni's multiple comparison test. Differences were accepted as statistically significant at *P < 0.05 or ^+^*P* < 0.05.

## RESULTS

### EFFECTS OF TF ON THE EXTRACELLULAR ATP RELEASE

**Fig 1:**
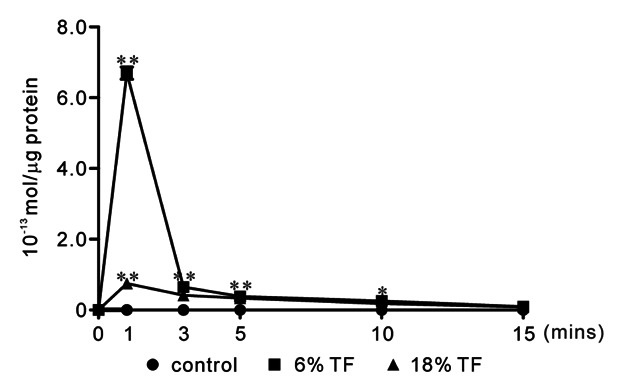
Time course of ATP release by TF stimulation. MC3T3-E1 cells were cultured with ATPase inhibitor ARL67156 (50 μM) for 30 min before TF stimulation and stimulated by 0% (control), 6%, or 18% TF for 0, 1, 5, 10, or 15 min. ATP release was determined by Luciferin-luciferase bioluminescence assay. Data are expressed as the mean ± standard error (SEM), n = 3 independent experiments. *P < 0.05, **P < 0.01, TF stimulation vs. control.

We investigated the effect of TF stimulation on extracellular ATP release in osteoblasts. Both 6% and 18% TF stimulation enhanced extracellular ATP release in the culture media compared to control at 1 min, respectively. Six percent TF enhanced the extracellular ATP release compared to 18% TF (Fig. 1).

### EFFECTS OF TF ON THE EXPRESSION OF Runx2, OSTERIX, AND P2X7 RECEPTOR

**Fig 2:**
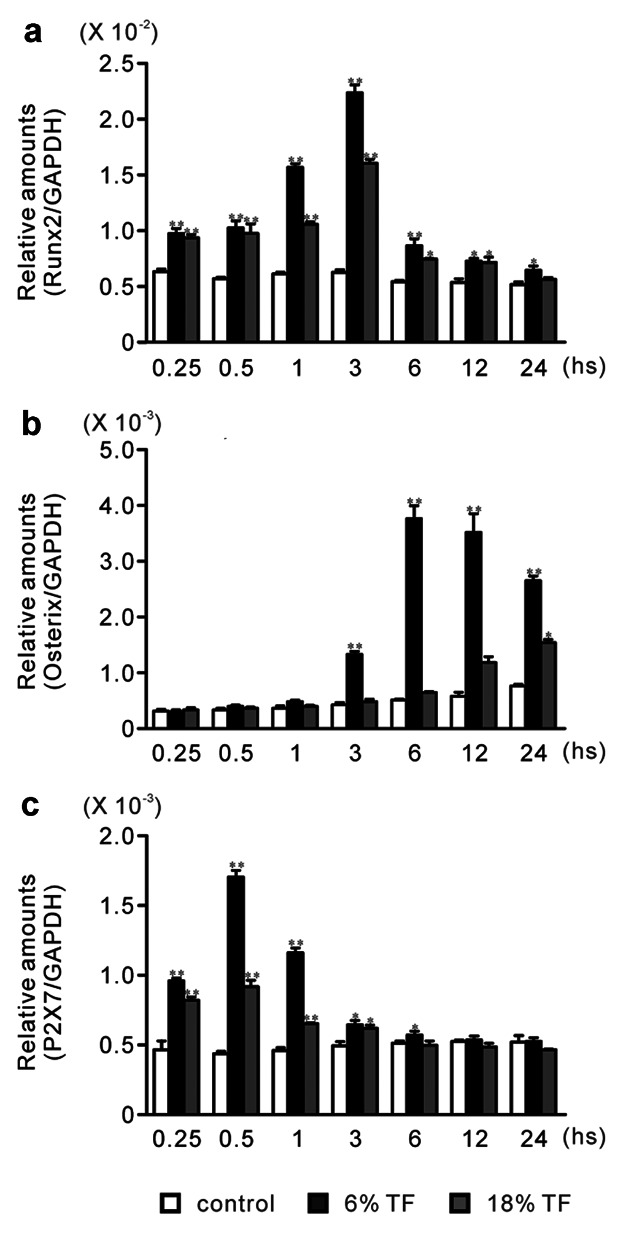
Effects of TF stimulation on gene expression of Runx2, Osterix, and P2X7 receptor. Cells were stimulated with 0% (control), 6%, or 18% TF for up to 24 h. The mRNA expression of Runx2 (a), Osterix (b), and P2X7 (c) was determined using real-time PCR at hours 0.25, 0.5, 1, 3, 6, 12, and 24 of culture. Data are shown as the mean ± SEM, n = 3 independent experiments. *P < 0.05, **P < 0.01, TF stimulation vs. control.

In both 6% and 18% TF stimulation, the mRNA expression of Runx2 gradually increased through hour 3 of culture and decreased by hour 24 (Fig. 2a). The expression of Runx2 at 6% and 18% TF stimulation was significantly increased by 1.4–3.6 and 1.2–2.5-fold compared to control, respectively, at hours 0.25, 0.5, 1, 3, 6, 12, and/or 24 of culture.

In 6% TF stimulation, the mRNA expression of Osterix gradually increased through hour 6 of culture and decreased by hour 24 (Fig. 2b). On the other hand, the expression at 18% TF stimulation gradually increased through hour 24 of culture. The expression of Osterix at 6% and 18% TF stimulation was significantly increased by 3.3–9.0 and 2.1-fold, respectively, compared to control at hours 3, 6, 12, and/or 24 of culture.

In both 6 and 18% TF stimulation, the mRNA expression of P2X7 receptor gradually increased through hour 0.5 of culture and decreased by hour 24 (Fig. 2c). The expression of P2X7 receptor at 6% and 18% TF stimulation was significantly increased by 1.2–4.1 and 1.2–2.2-fold compared to control, respectively, at hours 0.25, 0.5, 1, 3, and/or 6 of culture.

### EFFECTS OF A348079 AND/OR 6% TF ON Runx2 AND OSTERIX EXPRESSIONS

In order to clarify participation of TF-induced ATP in the increased Runx2 and Osterix expressions, we investigated the effect of P2X7 receptor selective antagonist A348079 [McGaraughty et al., 2007; Donnelly-Roberts et al., 2008] and/or 6% TF stimulation on Runx2 expression at 3 h and Osterix expression at 6 h. We decided to use 6% TF because it had stronger effects compared to 18% TF according to Figure 2. In addition the mRNA expression of Runx2 and Osterix showed the highest value at 3 and 6 h, respectively, as shown in Figure 2a and b.

**Fig 3:**
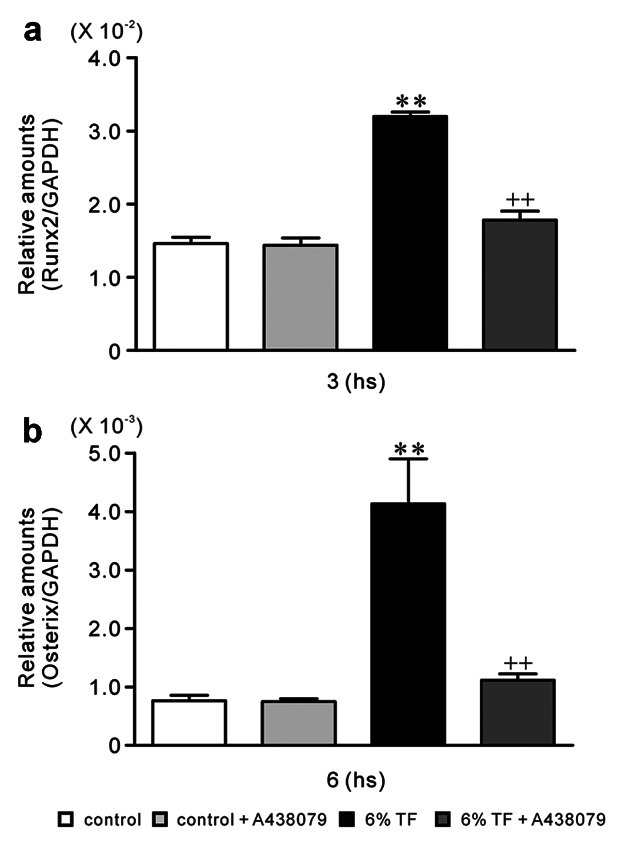
Effects of A438079 and/or 6% TF stimulation on gene expression of Runx2 and Osterix. Cells were cultured with or without 6% TF stimulation in the presence or absence of A438079 (10 μM). The mRNA expression of Runx2 (a) and Osterix (b) was determined using real-time PCR at hours 3 or 6 of culture, respectively. Data are shown as the mean ± SEM, n = 3 independent experiments.*P < 0.05, **P < 0.01, TF stimulation vs. control, ^++^*P* < 0.01, 6% TF stimulation vs. 6% TF stimulation + A438079.

**Fig 4:**
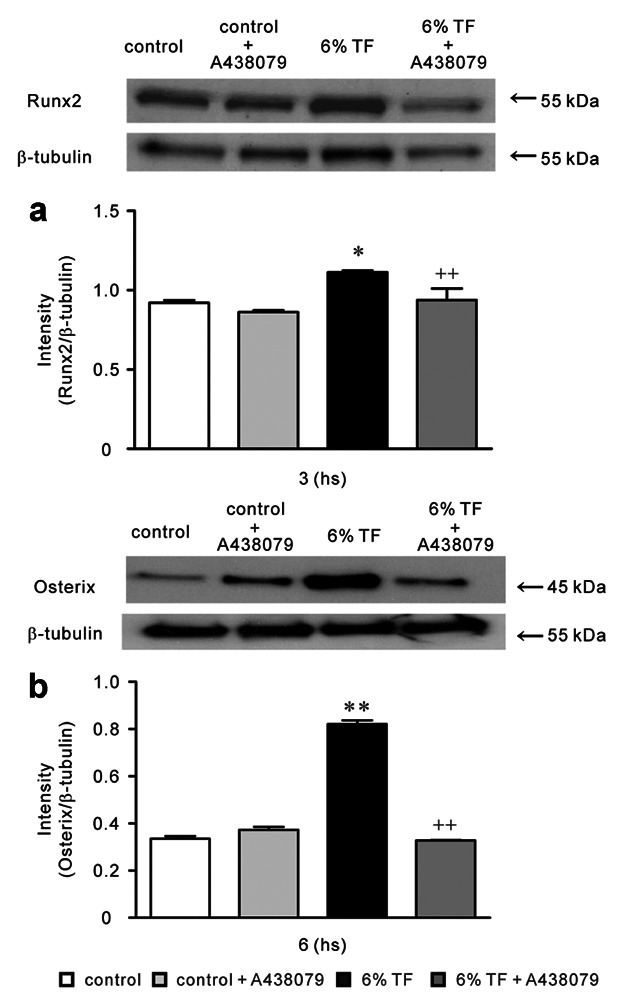
Effects of A438079 and/or 6% TF stimulation on protein expression of Runx2 and Osterix. Cells were cultured with or without 6% TF stimulation in the presence or absence of A438079 (10 μM). The protein expression of Runx2 (a) and Osterix (b) was determined using Western blotting on hours 3 or 6 of culture, respectively. Data are shown as the mean ± SEM, n = 3 separate experiments. *P < 0.05, **P < 0.01, TF stimulation vs. control, ^++^*P* < 0.01, 6% TF stimulation vs. 6% TF stimulation + A438079.

A438079 blocked the stimulatory effect of 6% TF stimulation on Runx2 and Osterix expressions at mRNA and protein levels (Figs. 3 and 4).

### EFFECTS OF A438079 AND/OR 6% TF ON THE EXPRESSION OF ECMPs

To investigate the role of 6% TF-induced ATP on osteogenesis, we determined the expressions of ECMPs at mRNA and protein levels in the presence of A438079 and/or 6% TF.

**Fig 5:**
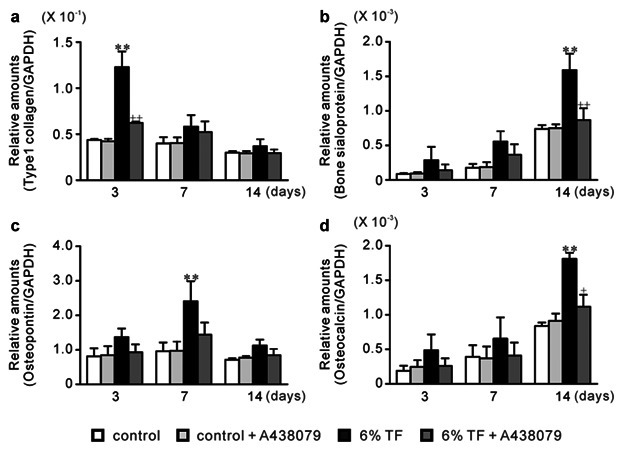
Effects of A438079 and/or 6% TF stimulation on gene expression of ECMPs. Cells were stimulated with or without 6% TF stimulation for 3 h/day, and cultured in the presence or absence of A438079 (10 μM) for up to 14 days. The mRNA expression of type I collagen (a), BSP (b), OPN (c), and OCN (d) was determined by real-time PCR on days 3, 7, and 14 of culture. Data are shown as the mean ± SEM, n = 3 independent experiments.*P < 0.05, **P < 0.01, TF stimulation vs. control, ^+^*P* < 0.05, ^++^*P* < 0.01, 6% TF stimulation vs. 6% TF stimulation + A438079.

**Fig 6:**
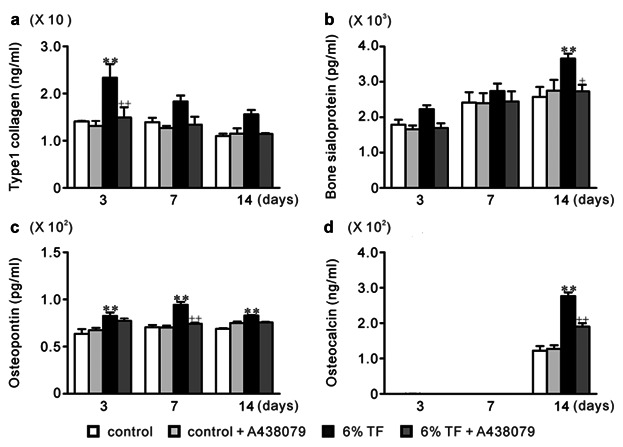
Effects of A438079 and/or 6% TF stimulation on protein expression of ECMPs. Cells were stimulated with or without 6% TF stimulation for 3 h/day, and cultured in the presence or absence of A438079 (10 μM) for up to 14 days. The protein expression of type I collagen (a), BSP (b), OPN (c), and OCN (d) was determined by ELISA on days 3, 7, and 14 of culture. Data are shown as the mean ± SEM, n = 3 independent experiments. **P < 0.01, TF stimulation vs. control, ^+^*P* < 0.05, ^++^*P* < 0.01, 6% TF stimulation vs. 6% TF stimulation + A438079.

In 6% TF stimulation, the mRNA expression of type I collagen, bone sialoprotein (BSP), osteopontin (OPN), and osteocalcin (OCN) was increased by 2.8-, 2.2-, 2.7-, and 2.2-fold, respectively, compared to control on days 3, 7, and/or 14 of culture (Fig. 5). Protein expressions were also increased by 6% TF stimulation as well as mRNA expressions (Fig. 6). A438079 blocked the stimulatory effect of 6% TF stimulation on each ECMP expression at mRNA and protein levels (Figs. 5 and 6).

### EFFECTS OF A438079 AND/OR 6% TF ON ALP ACTIVITY AND CALCIUM CONTENT IN ECM

**Fig 7:**
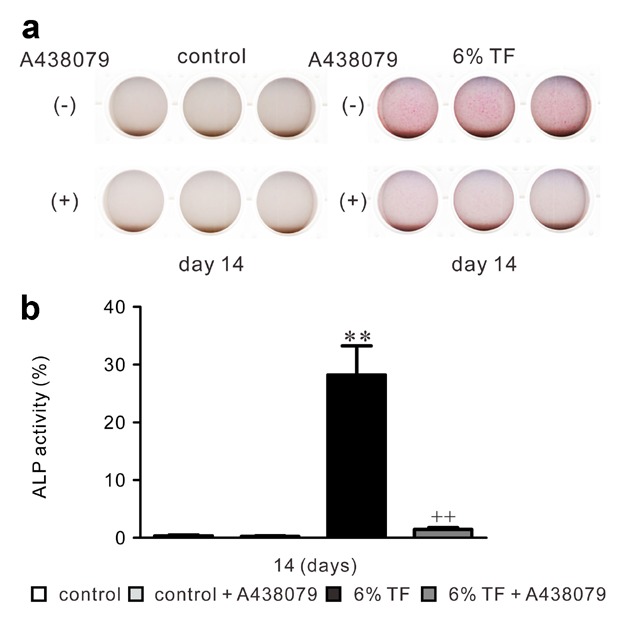
Effects of A438079 and/or 6% TF stimulation on ALP activity. Cells were stimulated with or without 6% TF stimulation for 3 h/day, and cultured in the presence or absence of A438079 (10 μM) for 14 days. Cells were stained by ALP activity staining. Cells were observed under a phase-contrast microscope (original magnification, ×100) (a). Histograms showed the percentage of ALPase activity under each condition (b). Data are shown as the mean ± SEM, n = 3 independent experiments. **P < 0.01, TF stimulation vs. control, ^+^*P* < 0.05, ^++^*P* < 0.01, 6% TF stimulation vs. 6% TF stimulation + A438079.

**Fig 8:**
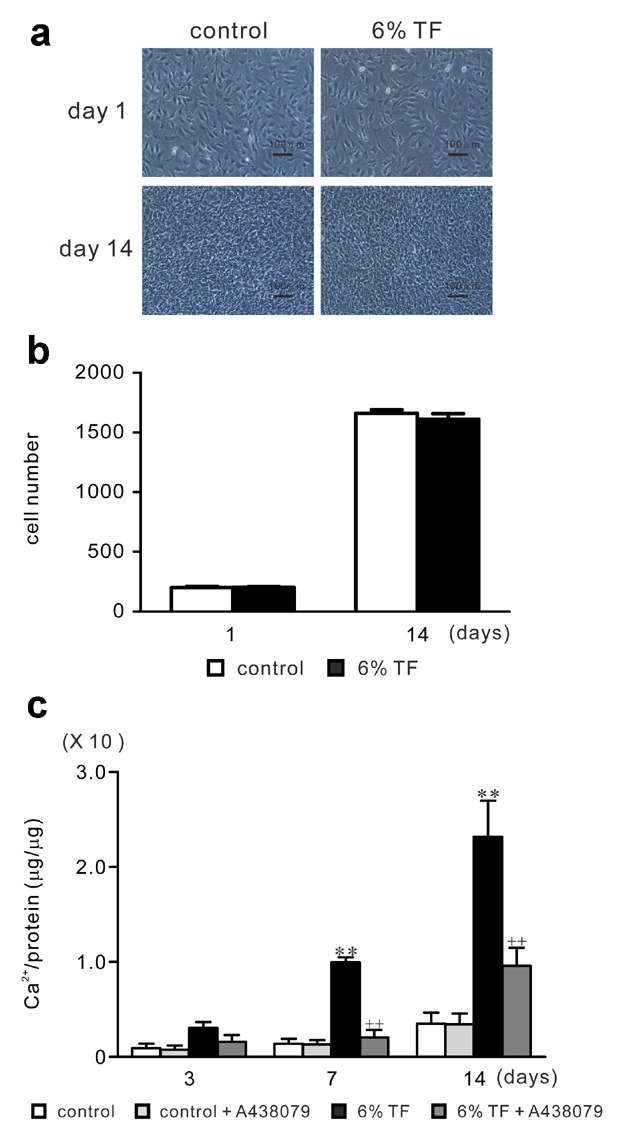
Effects of A438079 and/or 6% TF stimulation on calcium content in extracellular matrix. Cells were stimulated with or without 6% TF stimulation for 3 h/day, and cultured in the presence or absence of A438079 (10 μM) for up to 14 days. Cell were stained using 0.4 w/v% Trypan blue solution on days of 1 and 14 culture. Histograms showed the percent cell viability under each condition (a, b). Cells were observed under a phase-contrast microscope (original magnification, ×100) (a). Histograms showed the number of the cells under each condition (b). Calcium content in extracellular matrix was quantified by Calcium E-Test Wako (c). Data are shown as the mean ± SEM, n = 3 independent experiments. **P < 0.01, TF stimulation vs. control, ^++^*P* < 0.01, 6% TF stimulation vs. 6% TF stimulation + A438079.

To investigate the role of 6% TF-induced ATP in mineralized nodule formation, we examined ALP activity and calcium content of ECM in the presence of A438079 and/or 6% TF stimulation on day 14 of culture. ALP activity was enhanced by 6% TF stimulation compared to control (Fig. 7). In contrast, A438079 reduced the stimulatory effect of 6% TF stimulation on ALP activity. 6% TF stimulation did not affect cell viability on 1 and 14 days of culture (Figs. 8a and b). Calcium content of ECM was increased by 6.7-fold in 6% TF stimulation compared to control on days 7 and 14 of culture (Fig. 8b). A438079 reduced the stimulatory effect of 6% TF stimulation on the calcium content.

## DISCUSSION

Mechanical stress, such as orthodontic tooth movement [Roberts et al., 1981], increases bone resorption on the compression side and bone formation on the tension side [King et al., 1997]. In previous studies, compressive force (CF) induced not only osteoblast differentiation via PGE_2_ production [Mitsui et al., 2006] but also many kinds of inflammatory cytokines and mediators that induce osteoclast differentiation [Sanuki et al., 2007].

Bone cells stimulate the release of extracellular ATP release via a constitutive and inductive mechanism [Gartland et al., 2003; Genetos et al., 2005]. Komarova et al. [2000] reported that extracellular ATP release stimulates osteogenesis in vitro. The authors also reported that a fivefold increases in cytosolic ATP content correlated with the differentiation of rat calvarial cells.

ATP and 2′(3)-O-(4-benzoylbenzoyl)adenosine-5′-triphosphate (BzATP) induce the opening of the P2X7 nonselective cation channel which is permeable to Na^+^, K^+^, and Ca^2+^. The activation of P2X7 has been reported to causes an elevation in [Ca^2+^]_i_ and the depolarization of the plasma membrane [Pelegrin and Surprenant, 2006]. Elevated concentrations of ATP or BzATP have been found to facilitate the uptake of ethidium bromide in osteoblasts cultured in a divalent cation-free buffer [Gartland et al., 2001]. Furthermore, osteoblastic cells have been reported to express a functional P2X7 receptor in situ [Panupinthu et al., 2008]. Therefore, we investigated the effects of TF on P2X7 receptor-mediated differentiation and osteogenesis in osteoblastic cell line MC3T3-E1 osteoblast cells. We observed that TF stimulation for 1 min had the maximal effect on ATP release, and P2X7 receptor mRNA expression in MC3T3-E1 cells. In osteoblast cultures, the half-life of endogenously-released extracellular ATP is ∼10 min [Orriss et al., 2009]. Moreover, the effect of its downstream of signals are likely to be longer lasting [Burnstock, 2007]. Extracellular ATP concentrations depend on the balance between ATP release and its degradation by ectonucleotidases. In P2rx7^−/−^ mice, osteogenesis [Ke et al., 2003] and the anabolic responses to mechanical loading [Li et al., 2005] reduced, suggesting that ATP release into the bone extracellular fluid occurred in vivo. These studies suggest that constitutive ATP release in vivo is insufficient to activate P2X7 receptor. In contrast, ATP release induced by mechanical loading could trigger P2X7 signaling. In addition, 6% TF stimulation significantly enhanced the expression of the osteoblast differentiation-related transcriptions factors-Runx2 and Osterix. P2X7 receptor selective antagonist A438079 [McGaraughty et al., 2007; Donnelly-Roberts et al., 2008] blocked the expression of Runx2 and Osterix induced by 6% TF stimulation. Runx2 and Osterix are master regulators of osteoblast differentiation, as assessed by the characterization of mice lacking the genes encoding Runx2 and Osterix [Ducy et al., 1997; Nakashima et al., 2002]. Runx2 functions upstream of Osterix and regulates its expression [Nishio et al., 2006]. A438079 blocked BzATP mediated changes in intracellular calcium concentration via rat P2X7 receptor but not other P2 receptors [McGaraughty et al., 2007; Donnelly-Roberts et al., 2008]. A438079 could also inhibit mechanical and thermal inputs to several different classes of spinal neurons [McGaraughty et al., 2007]. In this study, we observed that the peak expression of Runx2 occurred earlier than that of Osterix, which was consistent with previous studies. Moreover, Nakashima et al. [2002] reported that the gene encoding OCN, which is regulated by Osterix, was downregulated in P2X7^−/−^ calvarial cell cultures.

Type I collagen is a major organic constituent of ECM in bone. It functions as the scaffold for the nucleation of hydroxyapatite crystals in during the calcification process. In contrast, non-collagenous matrix proteins play important roles in the organization of the collagen matrix and in regulating the formation and growth of hydroxyapatite crystals [Boskey, 1992]. The major non-collagenous proteins BSP, OPN and OCN are the chondroitin-sulfate glycoproteins in bone [Sodek et al., 1991]. BSP is a glycosylated and sulfated phosphoprotein found almost exclusively in mineralized connective tissues. During the bone formation process, BSP becomes the nucleus for hydroxyapatite formation [Hunter and Goldberg, 1993]. OPN which is a highly phosphorylated sialoprotein is a prominent component of mineralized bone ECM [Oldberg et al., 1986]. The functional diversity of OPN in bone formation and remodeling is related to its fundamental roles in host defense and tissue repair [Sodek et al., 2000]. OCN is the most abundant non-collagenous bone matrix protein [Wolf, 1996]. It is a small γ-carboxyglutamate protein that is expressed preferentially by osteoblasts and can bind to calcium ions [Boivin et al., 1990]. ALP, hydrolyzes ester bonds in organic phosphate compounds under alkaline conditions and plays an important role in bone calcification [Anderson, 1989]. High ALP activity correlated with extracellular matrix formation in osteoblasts before the initiation of mineralization [Gerstenfeld et al., 1987]. In this study, we observed that stimulation with 6% TF enhanced ECMPs expression, ALP activity, and calcium content in the ECM. Moreover, A438079 blocked the stimulatory effects of 6% TF stimulation on osteogenesis.

There are several conflicting reports describing potential roles for P2X7 in bone formation. Ke et al. [2003] reported that P2X7^−/−^ decreased bone mineral content and periosteal circumference, which was accompanied by reduced bone formation and enhanced bone resorption parameters. In contrast, Gartland et al. [2003] reported that P2X7^−/−^ did not affect bone mineral density in the trabecular bone, but promoted expansion in the cortical bone. These two reports are conflicting; however, an additional study reported that P2X7 knockout model can express a P2X7 splice variant in some tissues [Nicke et al., 2009]. Polymorphisms in the P2X7 receptor were correlated with an increased fracture risk in humans [Ohlendorff et al., 2007]. Orriss et al. reported that lack of the P2X7 receptor was expected to induce, rather than reduce, bone mass, but that both collagen production and adipocyte formation were unaffected [Orriss et al., 2012, 2013]. However, this study was performed in rat, rather than human or mouse, osteoblasts. In contrast, BzATP stimulated pore formation in cells within the nodules on mouse and rat osteoblasts. Furthermore, exogenous nucleotides activated P2X7 receptor and then, stimulated osteoblast differentiation and induced mineralization. On the other hand, P2rx7^−/−^ mice suppressed the expression of osteoblast markers in calvarial cells, compared to that in wild-type controls [Panupinthu et al., 2008]. These results suggest that the functional P2X7 receptor promotes osteogenesis in calvarial cells in vitro [Panupinthu et al., 2008]. Our present study is consistent with the in vivo studies published recently. The available data suggest that the P2X7 receptor (expresses several splice variants and single nucleotide polymorphisms and is highly complex [Gartland et al., 2003]). Furthermore, species-specific effects may occur.

Panupinthu et al. [2008] reported that P2X7 receptor agonist BzATP promotes the production of phosphatidic acid—through the activation of phospholipase D and promotes the production of lysophosphatidic acid via the activation of PLD and phospholipase A_2_ (LPA) Therefore, these pathways, including the cyclooxygenase (COX) pathway, may play a role in P2X7-induced osteogenesis in concert with LPA receptor signaling in osteoblasts. COXs mediated the conversion of arachidonic acid into prostaglandin (PG) E_2_. ATP release in response to mechanical stimulation induces the production of PGE_2_ in MC3T3-E1 cells [Genetos et al., 2005]. PGE_2_ production induced by mechanical stimulation decreases in the osteoblasts of P2rx7^−/−^ mice [Li et al., 2005]. Furthermore, PGE_2_ stimulates BSP transcription via juxtaposed CRE and FRE elements in the proximal promoter of the BSP gene in UMR 106 cells [Samoto et al., 2003]. We found that 6% TF stimulation induces PGE_2_ production (data not shown). These observations are consistent with the hypothesis, that 6% TF-induced PGE_2_ stimulated the expression of BSP. This finding suggests that PGE_2_ produced by the activation of P2X7 receptor act as an autocrine factor in osteoblasts. We plan to investigate the interaction between PGE_2_, TF stimulation, and P2X7 receptor in osteogenesis in a future study.

Our results suggest that 6% TF stimulation-induced ATP release in osteoblasts. In addition, 6% TF stimulation promotes osteogenesis via P2X7 receptor in osteoblasts. Therefore, we conclude that the activation of P2X7 by TF-induced-ATP is likely to play a critical role in bone formation.
